# miRNA-337-3p inhibits gastric cancer progression through repressing myeloid zinc finger 1-facilitated expression of matrix metalloproteinase 14

**DOI:** 10.18632/oncotarget.9739

**Published:** 2016-05-31

**Authors:** Liduan Zheng, Wanju Jiao, Hong Mei, Huajie Song, Dan Li, Xuan Xiang, Yajun Chen, Feng Yang, Huanhuan Li, Kai Huang, Qiangsong Tong

**Affiliations:** ^1^ Department of Pathology, Union Hospital, Tongji Medical College, Huazhong University of Science and Technology, Wuhan 430022, Hubei Province, P. R. China; ^2^ Clinical Center of Human Genomic Research, Union Hospital, Tongji Medical College, Huazhong University of Science and Technology, Wuhan 430022, Hubei Province, P. R. China; ^3^ Department of Pediatric Surgery, Union Hospital, Tongji Medical College, Huazhong University of Science and Technology, Wuhan 430022, Hubei Province, P. R. China

**Keywords:** gastric cancer, microRNA-337-3p, myeloid zinc finger 1, matrix metalloproteinase 14, transcriptional regulation

## Abstract

Matrix metalloproteinase 14 (MMP-14), a membrane-anchored MMP that promotes the tumorigenesis and aggressiveness, is highly expressed in gastric cancer. However, the transcriptional regulators of MMP-14 expression in gastric cancer still remain largely unknown. In this study, through mining computational algorithm programs and chromatin immunoprecipitation datasets, we identified adjacent binding sites of myeloid zinc finger 1 (MZF1) and miRNA-337-3p (miR-337-3p) within the *MMP-14* promoter. We demonstrated that MZF1 directly bound to the *MMP-14* promoter to facilitate its nascent transcription and expression in gastric cancer cell lines. In contrast, endogenous miR-337-3p suppressed the MMP-14 expression through recognizing its binding site within *MMP-14* promoter. Mechanistically, miR-337-3p repressed the binding of MZF1 to *MMP-14* promoter via recruiting Argonaute 2 and inducing repressive chromatin remodeling. Gain- and loss-of-function studies demonstrated that miR-337-3p suppressed the growth, invasion, metastasis, and angiogenesis of gastric cancer cells *in vitro* and *in vivo* through repressing MZF1-facilitated MMP-14 expression. In clinical specimens and cell lines of gastric cancer, MZF1 was highly expressed and positively correlated with MMP-14 expression. Meanwhile, miR-337-3p was under-expressed and inversely correlated with MMP-14 levels. miR-337-3p was an independent prognostic factor for favorable outcome of gastric cancer, and patients with high MZF1 or MMP-14 expression had lower survival probability. Taken together, these data indicate that miR-337-3p directly binds to the *MMP-14* promoter to repress MZF1-facilitatd MMP-14 expression, thus suppressing the progression of gastric cancer.

## INTRODUCTION

Gastric cancer is one of the most common cancers worldwide, with approximately 1 million new cases being diagnosed annually [1]. The prognosis of advanced gastric cancer remains poor, mainly due to tumor recurrence, invasion and metastasis, with a 5-year survival rate below 30% [1]. Better elucidating the mechanisms of tumorigenesis and aggressiveness is important for improving the therapeutic efficiency of gastric cancer [2]. Matrix metalloproteinase 14 (MMP-14), a membrane-anchored MMP, is not only able to degrade structural extracellular matrix (ECM) components such as fibronectin, vitronectin, laminin-1, laminin-5, fibrin, and collagen, but also capable of activating MMP-2, thereby promoting tumor invasion and metastasis [3]. In addition, MMP-14 contributes to pro-angiogenic responses through facilitating the expression of vascular epidermal growth factor (*VEGF*) and releasing bioactive ECM products [4]. High MMP-14 expression has been documented in most types of human malignancies, including colon cancer [5], breast cancer [6], ovary cancer [7], and skin cancer [8], and its elevated expression is associated with tumor invasion and metastasis [9]. Previous studies have shown that MMP-14 is highly expressed in gastric cancer, and is correlated with poor outcome of patients [10], suggesting the importance of MMP-14 in the progression of gastric cancer.

Human *MMP-14* gene is localized at chromosome 14q11 and mainly regulated at the transcription level [11]. Transcription factors specificity protein 1, hypoxia-inducible factor 2 alpha, and Krüppel-like factor 8 have been identified as potent regulators of MMP-14 expression in prostate cancer, renal cell carcinoma, and breast cancer [11–13]. In ovarian cancer cells, polyomavirus enhancer activator 3 (PEA3) is able to induce MMP-14 expression via direct binding to its promoter, and knockdown of *PEA3* reduces the MMP-14 levels [14]. In addition, hepatocyte nuclear factor 4 alpha exhibits oncogenic activity through directly binding to the *MMP-14* promoter and facilitating its transcription in neuroblastoma [15]. However, the transcriptional regulators and underlying mechanisms essential for MMP-14 expression in gastric cancer are limitedly identified.

In the current study, through mining computational algorithm programs and chromatin immunoprecipitation (ChIP) datasets, we identified adjacent binding sites of myeloid zinc finger 1 (MZF1) and miRNA-337-3p (miR-337-3p) within the *MMP-14* promoter. We demonstrate, for the first time, that MZF1 is highly expressed and facilitates the transcription of *MMP-14* in gastric cancer. Meanwhile, miR-337-3p is under-expressed and anti-correlated with MMP-14 expression in clinical gastric cancer specimens. In addition, miR-337-3p directly binds to the *MMP-14* promoter to suppress its transcription via inducing chromatin remodeling and repressing MZF1 enrichment, thus inhibiting the growth, invasion, metastasis, and angiogenesis of gastric cancer cells *in vitro* and *in vivo*, suggesting the tumor suppressive functions of miR-337-3p in the progression of gastric cancer.

## RESULTS

### MZF1 facilitates the expression of MMP-14 in gastric cancer cell lines

**Figure 1:**
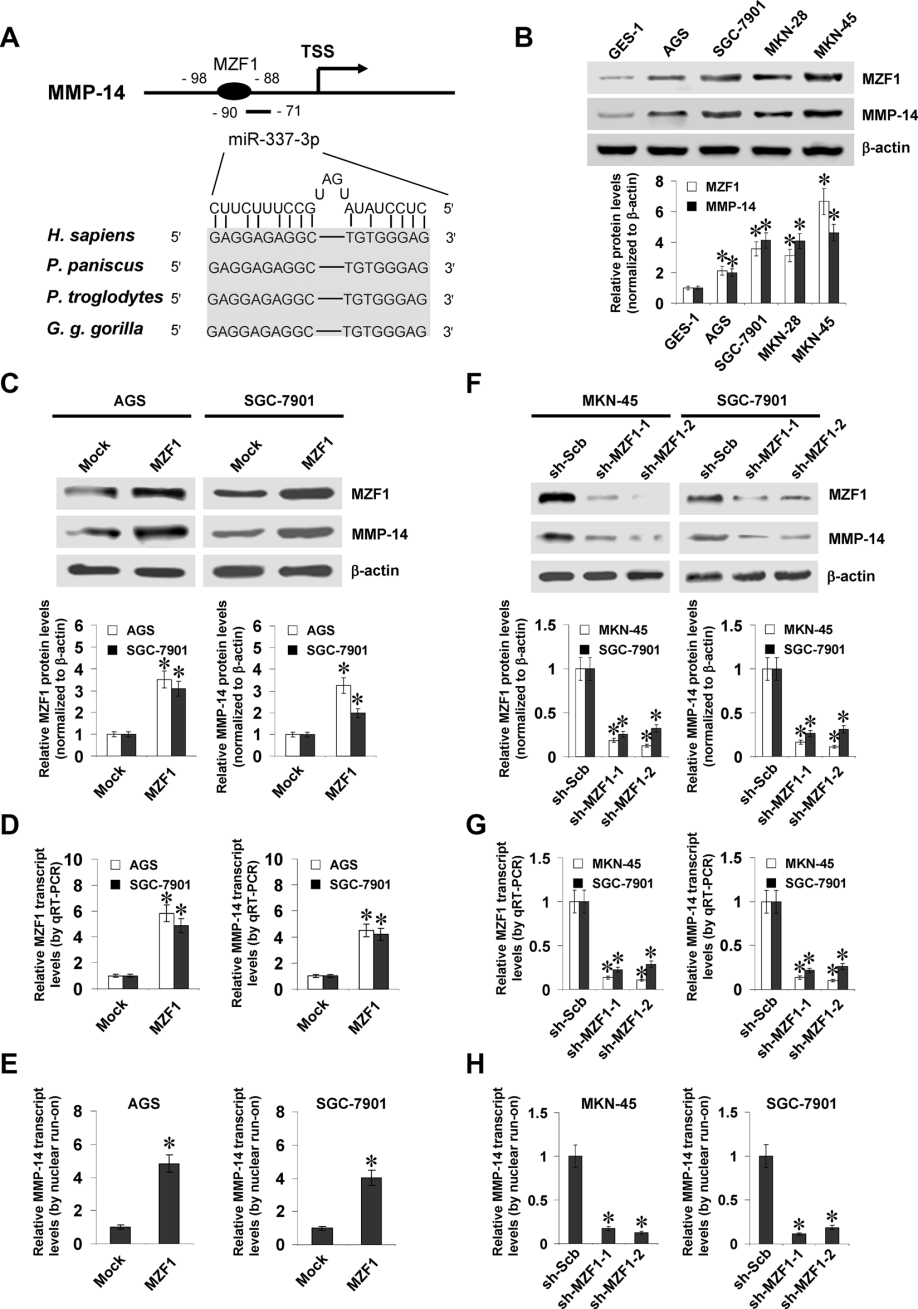
MZF1 facilitates the expression of MMP-14 in gastric cancer cells (**A**), scheme of potential binding sites of MZF1 and miR-337-3p within the *MMP-14* promoter, locating at bases −98/−88 and −90/−71 relative to TSS. (**B**), western blot showing the expression of MZF1 and MMP-14 in normal gastric epithelial GES-1 cells and gastric cancer cell lines (AGS, SGC-7901, MKN-28, and MKN-45). (**C** and **D**), western blot and real-time quantitative RT-PCR indicating the expression of MZF1 and MMP-14 in AGS and SGC-7901 cells stably transfected with empty vector (mock) or *MZF1*. (**E**) nuclear run-on assay showing the nascent *MMP-14* transcript levels in gastric cancer cells stably transfected with mock or *MZF1*. (**F** and **G**), western blot and real-time quantitative RT-PCR indicating the MZF1 and MMP-14 levels in MKN-45 and SGC-7901 cells stably transfected with scramble shRNA (sh-Scb) or *MZF1* shRNA (sh-MZF1). (**H**), nuclear run-on assay showing the nascent *MMP-14* transcript levels in gastric cancer cells transfected with sh-Scb or sh-MZF1. *P < 0.01 vs. GES-1, mock, or sh-Scb.

To investigate the mechanisms crucial for MMP-14 expression in gastric cancer, we first analyzed the activity of a series of *MMP-14* promoter fragments. Dual-luciferase assay indicated that −384/−95 bp relative to the transcription start site (TSS) was essential for the *MMP-14* promoter activity in cultured MKN-45 and SGC-7901 cells (Supplementary Figure S1A). Over-lapping analysis of computational algorithm programs Genomatrix [16], TFBIND [17], and PROMO [18] revealed the potential binding sites of MZF1, nuclear factor Y (NFY), and nuclear factor erythroid-2 related factor 2 (NRF2) within this region (chr14:23305444-23305733; Supplementary Figure S1B), locating at bases −98/−88, −158/−144, and −179/−159 relative to *MMP-14* TSS, respectively. Mining of publicly available ChIP-seq dataset [19] indicated the enrichment of MZF1, but not of NFY or NRF2, on *MMP-14* promoter region (Supplementary Figure S1B). Further analysis of Gene Expression Omnibus (GEO) datasets indicated the positive correlation between MZF1 and MMP-14 levels in different gastric cancer cohorts (Supplementary Figure S1B and S1C). In addition, the binding site of miR-337-3p with high complementarity was noted at −90/−71 bp region adjacent to that of MZF1 (Figure 1A). Higher MZF1 and MMP-14 levels were observed in gastric cancer cell lines than those in normal gastric epithelial cells (Figure 1B).

To address the hypothesis that MZF1 may influence the MMP-14 expression in gastric cancer cell lines, we performed the MZF1 over-expression and knockdown experiments. Western blot and real-time quantitative RT-PCR demonstrated that stable transfection of *MZF1* into AGS and SGC-7901 cells obviously increased the protein and transcript levels of MZF1 and MMP-14, than those stably transfected with empty vector (mock; Figure 1C and 1D). Nuclear run-on assay demonstrated that stable over-expression of *MZF1* increased the nascent transcript levels of *MMP-14* in gastric cancer cells, than those in mock cells (Figure 1E). On the other hand, short hairpin RNA (shRNA) specific for *MZF1* (sh-MZF1) was stably transfected into MKN-45 and SGC-7901 cells, resulting in decreased protein and transcript levels of MZF1 and MMP-14, when compared to those stably transfected with scramble shRNA (sh-Scb) (Figure 1F, 1G, and 1H). These results demonstrated that MZF1 considerably facilitated the MMP-14 expression at transcriptional levels in gastric cancer cells.

### MZF1 increases the transcription of *MMP-14* through direct binding to its promoter

**Figure 2:**
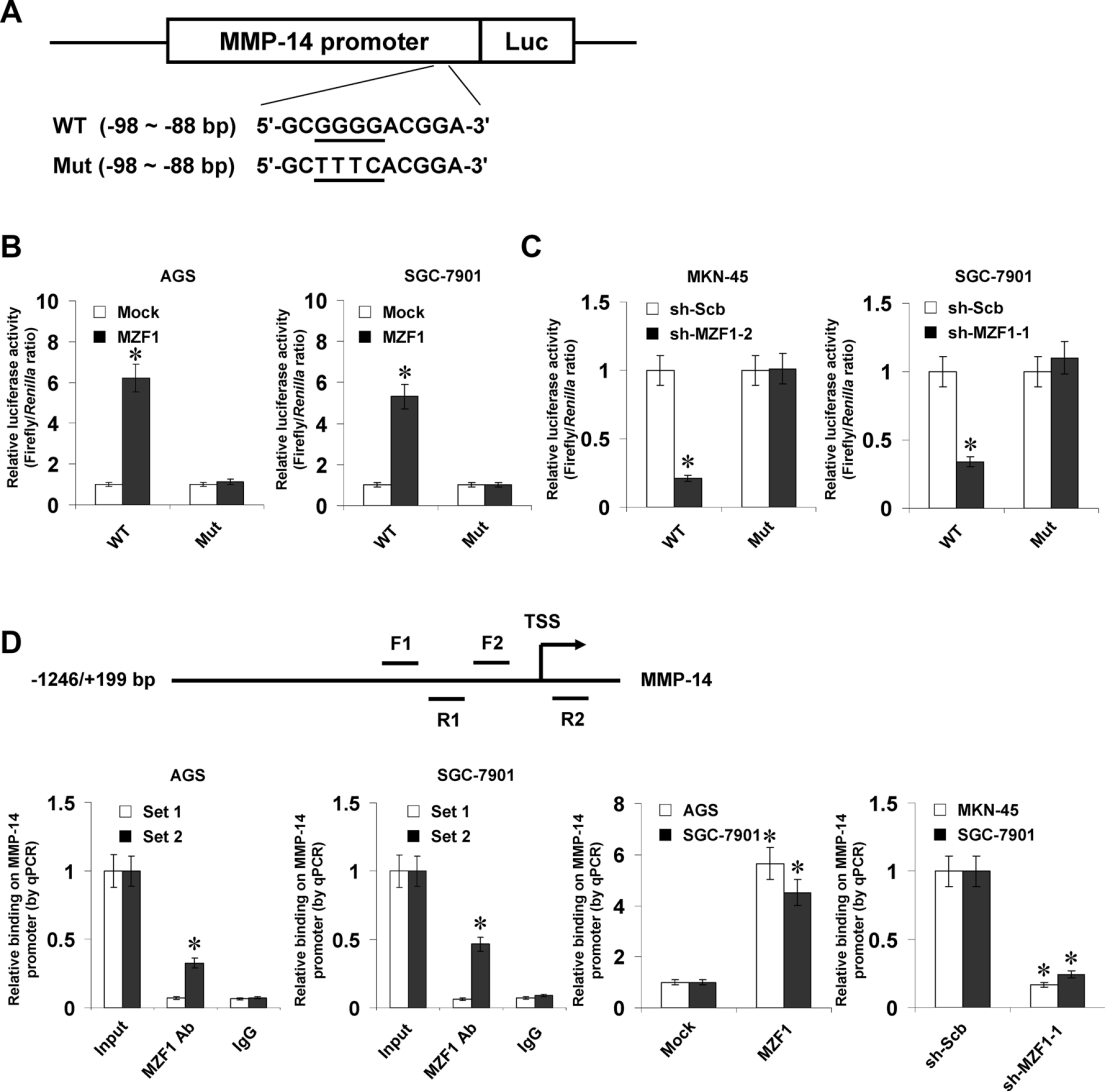
MZF1 increases the transcription of *MMP-14* through direct binding to its promoter (**A**), scheme and sequence of the intact MZF1 binding site (WT) and its mutation (Mut) within the *MMP-14* promoter-luciferase reporter vectors. (**B**), dual-luciferase assay showing the activity of *MMP-14* promoter and its mutant in AGS and SGC-7901 cells stably transfected with empty vector (mock) or *MZF1*. (**C**), dual-luciferase assay indicating the activity of *MMP-14* promoter and its mutant in MKN-45 and SGC-7901 cells stably transfected with scramble shRNA (sh-Scb) or *MZF1* shRNA (sh-MZF1). (**D**), ChIP and qPCR assay (normalized to input DNA) showing the enrichment of MZF1 on the *MMP-14* promoter in AGS, SGC-7901, and MKN-45 cells transfected with mock, *MZF1*, sh-Scb, or sh-MZF1. *P < 0.01 vs. mock, sh-Scb, or IgG.

To determine whether MZF1 could bind to the *MMP-14* promoter to increase its transcription, the *MMP-14* promoter luciferase reporter and its mutation vectors were transfected into gastric cancer cells (Figure 2A). Dual-luciferase assay indicated that ectopic expression or knockdown of *MZF1* enhanced and attenuated the promoter activity of *MMP-14* in gastric cancer cells, respectively (Figure 2B and 2C), and mutation of MZF1 binding site abolished these effects (Figure 2B and 2C). In addition, ChIP and real-time quantitative PCR (qPCR) were applied to measure the enrichment of MZF1 on *MMP-14* promoter with primer sets spanning its binding sites. In cultured AGS and SGC-7901 cells, enrichment of MZF1 was observed around its binding site (−122/+69 bp relative to TSS) (Figure 2D). As controls, no *MMP-14* promoter regions were immunoprecipitated with unspecific antibody (isotype IgG) or detected by qPCR with primer set (−326/−157 bp) distal to the binding site of MZF1 (Figure 2D). Stable transfection of *MZF1* into gastric cancer cells resulted in enrichment of MZF1 on the *MMP-14* promoter (Figure 2D). Meanwhile, stable knockdown of *MZF1* with shRNA construct decreased the binding of MZF1 to *MMP-14* promoter in MKN-45 and SGC-7901 cells (Figure 2D). These results indicated that MZF1 directly bound to the promoter of *MMP-14* to increase its transcription in gastric cancer cells.

### miR-337-3p recruits AGO2 to repress the transcription of *MMP-14*

**Figure 3:**
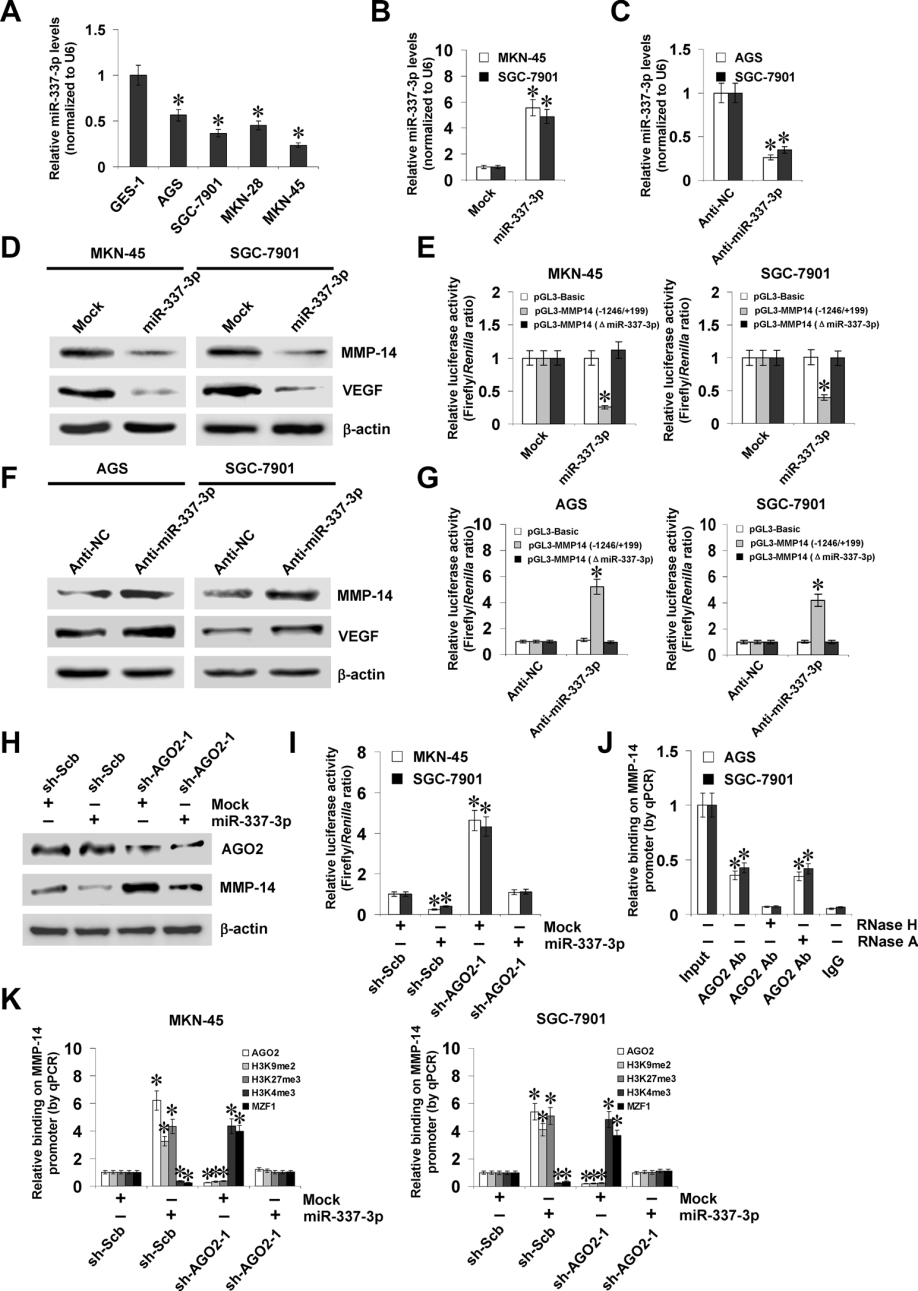
miR-337-3p recruits AGO2 to repress the transcription of *MMP-14* (**A**), real-time quantitative RT-PCR assay showing the miR-337-3p levels in normal gastric epithelial GES-1 cells and gastric cancer cell lines (AGS, SGC-7901, MKN-28, and MKN-45). (**B** and **C**), real-time quantitative RT-PCR assay indicating the miR-337-3p levels in gastric cancer cells transfected with empty vector (mock), miR-337-3p precursor, negative control inhibitor (anti-NC, 100 nmol/L), or anti-miR-337-3p inhibitor (100 nmol/L). (**D** and **E**), western blot and dual-luciferase assays showing the expression of MMP-14 and VEGF and promoter activity of *MMP-14* in MKN-45 and SGC-7901 cells stably transfected with mock or miR-337-3p precursor. (**F** and **G**), western blot and dual-luciferase assays indicating the expression of MMP-14 and VEGF and promoter activity of *MMP-14* in AGS and SGC-7901 cells transfected with anti-NC or anti-miR-337-3p inhibitor (100 nmol/L). (**H** and **I**), western blot and dual-luciferase assays showing the expression of AGO2 and MMP-14 and promoter activity of *MMP-14* in MKN-45 and SGC-7901 cells stably transfected with mock or miR-337-3p precursor, and those co-transfected with scramble shRNA (sh-Scb) or *AGO2* shRNA (sh-AGO2). (**J**) ChIP and qPCR assay indicating the binding of AGO2 to *MMP-14* promoter in gastric cancer cells treated with RNase H or RNase A. (**K**) ChIP and qPCR assay showing the enrichment of AGO2, H3K9me2, H3K27me3, H3K4me3, and MZF1 on *MMP-14* promoter in MKN-45 and SGC-7901 cells stably transfected with mock or miR-337-3p precursor, and those co-transfected with sh-Scb or sh-AGO2. *P < 0.01 vs. GES-1, mock, anti-NC, mock + sh-Scb, or IgG.

Since our previous studies have shown the functions of miR-337-3p in repressing *MMP-14* transcription [20], we observed the effects of miR-337-3p on MMP-14 expression in gastric cancer cells. Lower miR-337-3p expression was observed in gastric cancer cell lines than those in normal gastric epithelial cells (Figure 3A). Stable transfection of miR-337-3p precursor into MKN-45 and SGC-7901 cells increased the miR-337-3p levels (Figure 3B). Meanwhile, transfection of anti-miR-337-3p inhibitor obviously decreased the miR-337-3p levels in AGS and SGC-7901 cells (Figure 3C). Western blot, real-time quantitative RT-PCR, and dual-luciferase assays demonstrated that over-expression or knockdown of miR-337-3p decreased and increased the protein and transcript levels of MMP-14 in gastric cancer cells, than those transfected with empty vector (mock) or negative control (anti-NC) inhibitor, respectively (Figure 3D–3G, Supplementary Figure S2A and S2B). The expression of *VEGF*, the MMP-14 downstream gene in gastric cancer [21], was significantly decreased or increased in miR-337-3p over-expressing and knockdown gastric cancer cells, consistent with the MMP-14 levels (Figure 3D–3F, Supplementary Figure S2A and S2B). The analysis of microPIR database revealed no potential binding site of miR-337-3p within the *VEGF* promoter, ruling out the possibility that miR-337-3p may directly suppress the transcription of *VEGF*. Since previous studies have revealed the potential involvement of AGO2 in miR-337-3p-induced transcriptional repression [20], shRNAs against AGO2 were transfected into MKN-45 and SGC-7901 cells. Western blot, real-time quantitative RT-PCR, and dual-luciferase assays demonstrated that knockdown of *AGO2* abolished the miR-337-3p-induced transcriptional repression of *MMP-14* in gastric cancer cells (Figure 3H, 3I, Supplementary Figure S2C, and S2D). The *MMP-14* promoter region was detected for enrichment of AGO2 by ChIP and real-time qPCR. In cultured AGS and SGC-7901 cells, enrichment of AGO2 was observed around the binding site of miR-337-3p (Figure 3J). As controls, no *MMP-14* promoter regions were immunoprecipitated with unspecific antibody (isotype IgG) (Figure 3J). In addition, RNase H treatment, but not RNase A treatment, abolished the enrichment of AGO2 on the *MMP-14* promoter in gastric cancer cells (Figure 3J). Stable transfection of miR-337-3p precursor into gastric cancer cells resulted in increased binding of AGO2 and epigenetic markers histone H3 lysine 9 dimethylation (H3K9me2) and histone H3 lysine 27 trimethylation (H3K27me3), and decreased binding of histone H3 lysine 4 trimethylation (H3K4me3) and MZF1 on *MMP-14* promoter (Figure 3K), which were abolished by knockdown of *AGO2* (Figure 3K). Collectively, these results indicated that miR-337-3p recognized the binding site and recruited AGO2 to repress the *MMP-14* transcription in gastric cancer cells.

### miR-337-3p suppresses the growth, invasion, and angiogenesis of gastric cancer cells through repressing MZF1-facilitated MMP-14 expression *in vitro*

**Figure 4:**
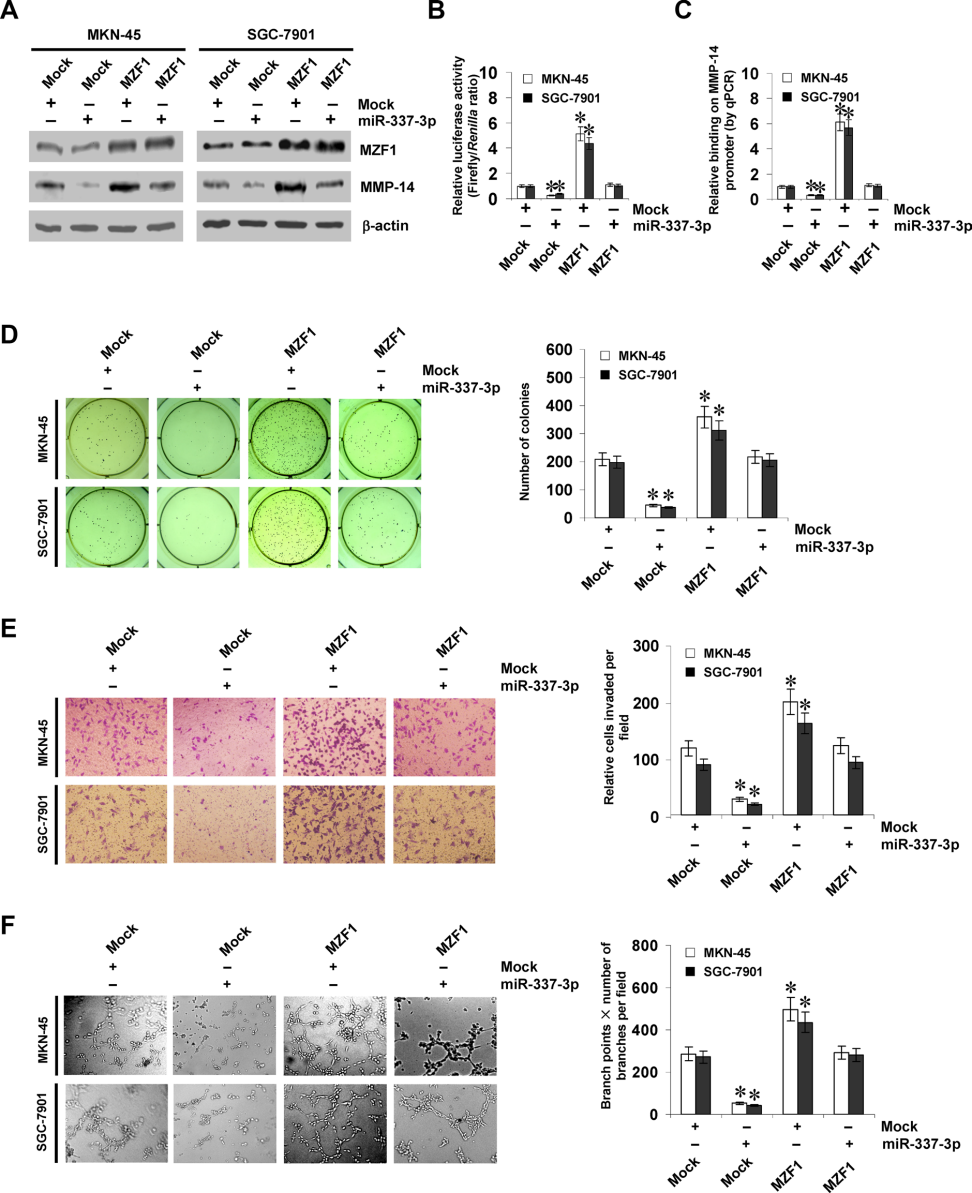
miR-337-3p suppresses the growth, invasion, and angiogenesis of gastric cancer cells through repressing MZF1-facilitated MMP-14 expression *in vitro* (**A** and **B**), western blot and dual-luciferase assays showing the expression of MZF1 and MMP-14 and promoter activity of *MMP-14* in MKN-45 and SGC-7901 cells stably transfected with empty vector (mock) or miR-337-3p precursor, and those co-transfected with *MZF1*. (**C**), ChIP and qPCR assay indicating the enrichment of MZF1 on *MMP-14* promoter in gastric cancer cells stably transfected with mock or miR-337-3p precursor, and those co-transfected with *MZF1*. (**D**), representation (left) and quantification (right) of soft agar assay showing the anchorage-independent growth of MKN-45 and SGC-7901 cells stably transfected with mock or miR-337-3p precursor, and those co-transfected with *MZF1*. (**E**), representation (left) and quantification (right) of matrigel invasion assay indicating the invasion capability of gastric cancer cells stably transfected with mock or miR-337-3p precursor, and those co-transfected with *MZF1*. (**F**), representation (left) and quantification (right) of tube formation assay showing the angiogenic capability of gastric cancer cells stably transfected with mock or miR-337-3p precursor, and those co-transfected with *MZF1*. *P < 0.01 vs. mock.

Since above evidence indicated that miR-337-3p attenuated the binding of MZF1 to *MMP-14* promoter, we further investigated the effects of miR-337-3p over-expression on MZF1-facilitated MMP-14 expression in gastric cancer cells. Western blot, real-time quantitative RT-PCR, and dual-luciferase assays indicated that ectopic expression of miR-337-3p abolished the enhanced protein and transcript levels of *MMP-14* induced by stable transfection of *MZF1* (Figure 4A, 4B, and Supplementary Figure S2E). In addition, over-expression of miR-337-3p prevented the enhanced MZF1 enrichment on *MMP-14* promoter induced by ectopic expression of *MZF1* (Figure 4C). In soft agar assay, MZF1 over-expression promoted the anchorage-independent growth of MKN-45 and SGC-7901 cells, when compared to those stably transfected with empty vector (mock; Figure 4D). In matrigel invasion assay, gastric cancer cells stably transfected with *MZF1* presented an increased invasion capacity than mock cells (Figure 4E). The tube formation of endothelial cells was increased by treatment with the medium preconditioned by stable transfection of gastric cancer cells with *MZF1* (Figure 4F). Moreover, transfection of miR-337-3p rescued the MKN-45 and SGC-7901 cells from increased growth, invasion, and angiogenesis capability induced by stable over-expression of *MZF1* (Figure 4D–4F). On the other hand, we examined the effects of *MZF1* knockdown on gastric cancer cells. Stable transfection of sh-MZF1 decreased the transcript and protein levels of MMP-14 (Supplementary Figure S2F, S2G, and S3A), and attenuated the capability of growth (Supplementary Figure S3B and S3C), invasion (Supplementary Figure S3D), and angiogenesis in AGS and SGC-7901 cells (Supplementary Figure S3E). Down-regulation of miR-337-3p via transfection of anti-miR-337-3p inhibitor rescued the AGS and SGC-7901 cells from their changes in these biological features induced by knockdown of *MZF1* (Supplementary Figure S3B–S3E). These results indicated that miR-337-3p significantly decreased the growth, invasion, and angiogenesis of gastric cancer cells through repressing MZF1-facilitated MMP-14 expression *in vitro*.

### miR-337-3p attenuates the growth, metastasis, and angiogenesis of gastric cancer cells through repressing MZF1-facilitated MMP-14 expression *in vivo*

**Figure 5:**
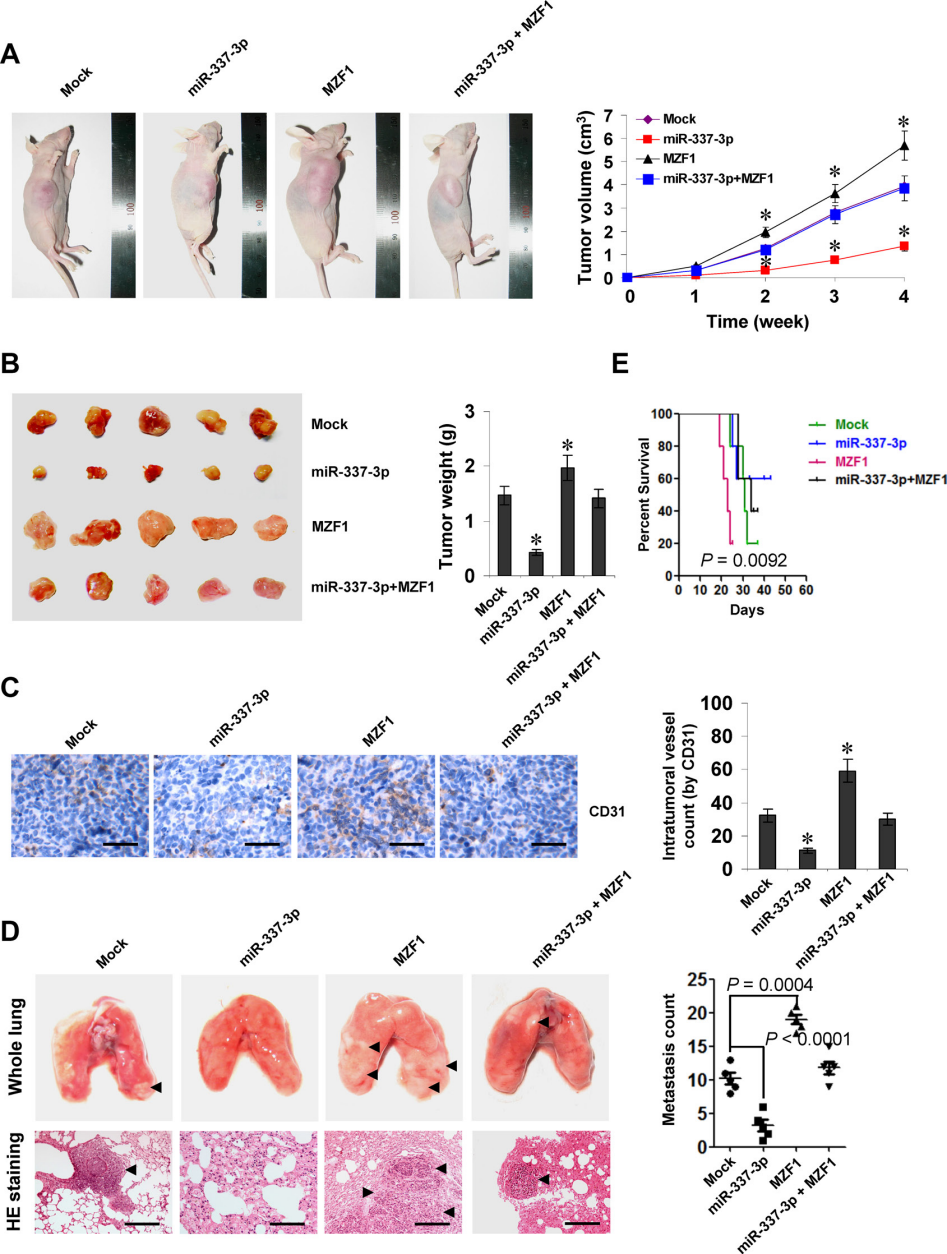
miR-337-3p attenuates the growth, metastasis, and angiogenesis of gastric cancer cells through repressing MZF1-facilitated MMP-14 expression *in vivo* (**A**), tumor growth curve of SGC-7901 cells (1 × 10^6^) stably transfected with empty vector (mock) or miR-337-3p precursor, and those co-transfected with *MZF1* in athymic nude mice (*n* = 5 for each group), after hypodermic injection for 4 weeks. (**B**), representation (left) and quantification (right) of xenograft tumors formed by hypodermic injection of SGC-7901 cells stably transfected with mock or miR-337-3p precursor, and those co-transfected with *MZF1*. (**C**), immunohistochemical staining (left) and quantification (right) of CD31 expression within tumors formed by hypodermic injection of SGC-7901 cells stably transfected with mock or miR-337-3p precursor, and those co-transfected with *MZF1*. Scale bars: 100 mm. (**D**), representation (left, arrowhead) and quantification (right) of lung metastasis after injection of SGC-7901 cells (0.4 × 10^6^) stably transfected with mock or miR-337-3p precursor, and those co-transfected with *MZF1* into the tail vein of athymic nude mice (*n* = 5 for each group). Scale bars: 100 mm. (**E**), Kaplan–Meier survival plots of nude mice with injection of SGC-7901 cells (0.4 × 10^6^) stably transfected with mock or miR-337-3p precursor, and those co-transfected with *MZF1* via the tail vein (*n* = 5 for each group). *P < 0.001 vs. mock.

We next investigated the efficacy of miR-337-3p against MZF1-facilitated tumor growth and metastasis *in vivo*. Stable transfection of *MZF1* into SGC-7901 cells resulted in increased growth and tumor weight of subcutaneous xenograft tumors in athymic nude mice, when compared to those stably transfected with empty vector (mock) (Figure 5A, 5B). In addition, stable transfection of *MZF1* resulted in increase in CD31-positive microvessels and mean vessel density within tumors (Figure 5C). In the experimental metastasis studies, SGC-7901 cells stably transfected with *MZF1* established statistically more lung metastatic colonies than mock group (Figure 5D), and resulted in lower survival probability of nude mice (Figure 5E). Moreover, stable transfection of miR-337-3p precursor into SGC-7901 cells prevented the MZF1-facilitated growth, metastasis and angiogenesis in athymic nude mice (Figure 5A–5E). These results suggested that miR-337-3p could attenuate the MZF1-facilitated growth, metastasis, and angiogenesis of gastric cancer cells *in vivo*.

### MZF1 and miR-337-3p are positively or inversely correlated with MMP-14 levels in gastric cancer tissues

**Figure 6:**
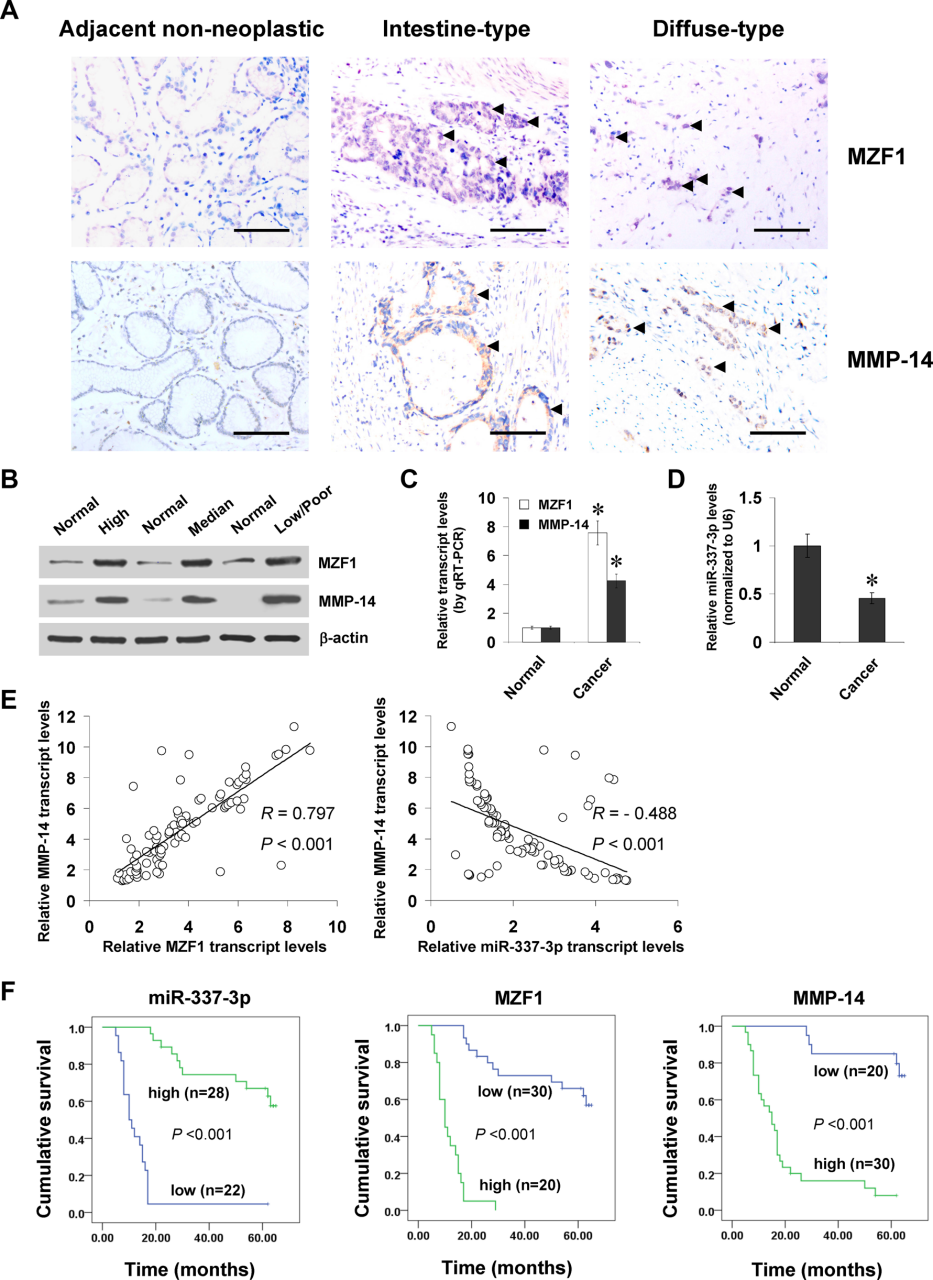
MZF1 and miR-337-3p are positively or inversely correlated with MMP-14 levels in gastric cancer tissues (**A**), immunohistochemical staining showing the expression of MZF1 and MMP-14 in the tumor cells of gastric cancer specimens (arrowheads, brown). Scale bars: 100 mm. (**B**), western blot assay indicating the protein levels of MZF1 and MMP-14 in gastric cancer tissues with different differentiation, and those in normal gastric mucosa. (**C** and **D**), real-time quantitative RT-PCR showing the transcript levels of *MZF1*, *MMP-14*, and miR-337-3p in normal gastric mucosa (*n* = 90) and gastric cancer tissues (*n* = 90). (**E**), the correlation between *MZF1* or miR-337-3p expression and *MMP-14* transcript levels in gastric cancer tissues (*n* = 90). (**F**), Kaplan–Meier survival plots of 50 well-defined gastric cancer cases with high or low expression of miR-337-3p, MZF1 or MMP-14. *P < 0.01 vs. normal.

To investigate the expression of MZF1 in gastric cancer tissues, paraffin-embedded sections from 50 well-established primary cases were collected. Immunohistochemical staining revealed that MZF1 was expressed in the nuclei of cancer cells (Figure 6A), and was detected in 30/50 (60.0%) cases, with weak staining in 10, moderate in 8, and intense in 12 (Supplementary Table S1). The MZF1 immunoreactivity was significantly higher in gastric cancer cases with deeper gastric wall invasion (*P* < 0.001), lymph node metastasis (*P* < 0.001), distant metastasis (*P* = 0.029), and advanced tumor-node-metastasis (TNM) stage (*P* < 0.001) (Supplementary Table S1). Notably, the immunostaining of MZF1 was associated with MMP-14 immunoreactivity in gastric cancer cases (correlation coefficient *R* = 0.500, *P* < 0.001, Figure 6A and Supplementary Table S2). Western blot and real-time quantitative RT-PCR were applied to measure the expression levels of MZF1, MMP-14, and miR-337-3p in 90 gastric cancer specimens and normal gastric mucosa. As shown in Figure 6B and 6C, higher protein and transcript levels of MZF1 or MMP-14 were observed in gastric cancer tissues than those in normal gastric mucosa, which was in line with the results from public datasets (Supplementary Figure S4A and S4B). In contrast, miR-337-3p was under-expressed in gastric cancer tissues than in normal gastric mucosa (Figure 6D and Supplementary Figure S4C). Notably, there was a positive correlation between *MZF1* and *MMP-14* transcript levels in gastric cancer tissues (correlation coefficient *R* = 0.797, *P* < 0.001, Figure 6E). Meanwhile, the miR-337-3p expression was inversely correlated with *MMP-14* transcript levels in gastric cancer tissues (*R* = − 0.488, *P* < 0.001, Figure 6E). Kaplan–Meier survival analysis revealed that patients with high miR-337-3p (*P* < 0.001) levels or low MZF1 (*P* < 0.001) and MMP-14 (*P* < 0.001) expression had greater survival probability than those with low or high expression, respectively (Figure 6F). Significant difference was noted among the survival curves of gastric cancer patients with low or high expression of miR-337-3p, MZF1, or MMP-14 (Supplementary Table S3). Cox regression analysis of these gastric cancer cases indicated that the distant metastasis (hazard ratio *HR* = 2.052, *P* = 0.002), TNM stage (*HR* = 2.722, *P* = 0.005), MZF1 expression (*HR* = 3.516, *P* = 0.018), miR-337-3p levels (*HR* = 0.393, *P* = 0.015), and MMP-14 expression (*HR* = 2.319, *P* = 0.028), but not patient's age (*HR* = 1.017, *P* = 0.765), gender (*HR* = 1.397, *P* = 0.675), tumor size (*HR* = 1.807, *P* = 0.467), Laurén classification (*HR* = 1.596, *P* = 0.545), tumor invasion (*HR* = 2.218, *P* = 0.332), or lymph node metastasis (*HR* = 2.018, *P* = .431), were independent prognostic factors for gastric cancer patients (Supplementary Table S4). These results indicated the up-regulation of MZF1 and under-expression of miR-337-3p in gastric cancer tissues, which was positively and inversely correlated with the MMP-14 levels, respectively.

## DISCUSSION

MZF-1, a transcription factor of Krüppel family proteins, was originally cloned from the cDNA library of chronic myeloid leukemia [22]. Recent evidence shows that MZF1 exerts oncogenic or tumor suppressive functions in different cell contexts. Over-expression of *MZF1* aggressively induces the transformation of NIH3T3 cells [23], and increases the migration and invasion of colorectal cancer cells [24], indicating the oncogenic functions of MZF1 in tumor progression. In contrast, MZF1 may also act as a tumor suppressor in the tumorigenesis. MZF1 inhibits the migratory and invasive capability of cervical cancer cells through reducing MMP-2 expression [25]. Knockout of *MZF1* increases the proliferative potentials of hemopoietic progenitors and results in lethal neoplasia in mice [26]. In this study, we demonstrated that MZF1 is highly expressed in gastric cancer, and is an independent prognostic factor for unfavorable outcome of patients. We found that knockdown of *MZF1* suppressed the growth, invasion, and angiogenesis of gastric cancer cells, suggesting the oncogenic functions of MZF1 during the progression of gastric cancer.

Human *MZF1* gene encodes a 485-amino acid protein that activates or suppresses gene transcription depending on the cellular environment [27]. MZF1 directly binds to the *p55PIK* promoter to activate its expression, and acts as a growth accelerator in colorectal cancer cells [24]. In lung adenocarcinoma cells, liver kinase B1 loss-induced MZF1 expression promotes the transcription of *c-Myc*, and is responsible for the growth, migration and invasion of cancer cells [28]. On the other hand, MZF1 directly binds to the promoters of *CD34* and *c-Myb* to suppress their transcription in hematopoietic cells [29]. However, the downstream genes of MZF-1 in gastric cancer still remain unknown. In this study, we demonstrated that *MMP-14* was a novel target gene of MZF1 in gastric cancer. First, the expression of MZF1 and MMP-14 was positively correlated in gastric cancer tissues and cell lines. Second, the activity of *MMP-14* promoter luciferase reporter was responsive to *MZF1* over-expression and knockdown. Third, mutation of MZF1 binding site abolished the regulatory effects of MZF1 on the *MMP-14* promoter luciferase reporter. Fourthly, ChIP and qPCR assay indicated the binding of MZF1 to *MMP-14* promoter. Finally, endogenous MMP-14 expression, both transcript and protein, was increased or decreased by over-expression and knockdown of *MZF1* in gastric cancer cells, suggesting that MZF1 may facilitate the MMP-14 expression by activating transcription.

Canonically, microRNAs (miRNAs), a class of small non-coding RNAs with 22 to 25 nucleotides in length, inhibit gene expression at the post-transcriptional levels [30]. Recent evidence shows that endogenous miRNAs are able to recognize complementary genomic sites within human gene promoters, and participate in the heterochromatin formation and regulation of gene transcription [31, 32]. For example, let-7 forms complex with AGO2 to repress the transcription of retinoblastoma 1/E2F downstream genes in senescence [33]. miR-709 is able to form epigenetic silencing complexes with H3K27me3 and AGO1, and represses the transcription of early growth response 2 [34]. In addition, miR-10a binds to the homologous region within homeobox D4 promoter to repress its expression at the transcriptional levels [35]. It has been indicated that miR-337-3p is under-expressed and associated with the lymph node metastasis of gastric cancer [36], and is able to directly bind to the *MMP-14* promoter to repress its transcription in neuroblastoma [20]. However, the functions and downstream gene of miR-337-3p in gastric cancer still remain to be elucidated. In this study, we noted the adjacent binding sites of miR-337-3p and MZF1 within *MMP-14* promoter. Our findings showed that miR-337-3p was an independent prognostic factor for favorable outcome of gastric cancer, and suppressed the expression of MMP-14 through repressing the enrichment of MZF1 on *MMP-14* promoter in gastric cancer cells. The fact that over-expression of miR-337-3p was sufficient to prevent the gastric cancer cells from MZF1-facilitated biological behaviors indicates that miR-337-3p exerts its tumor suppressive functions, at least in part, through repressing the MZF1 activity in gastric cancer.

Since AGO2 is essential for miRNA-directed implementation of silent-state chromatin modification at gene promoters [33], we further observed the functions of AGO2 in miR-337-3p-repressed MMP-14 expression in gastric cancer. We found that AGO2 was enriched surrounding the binding site of miR-337-3p within *MMP-14* promoter in gastric cancer cells. In addition, RNase H treatment (specifically degrades the RNA present in RNA-DNA hybrid) [32] attenuated the miR-337-3p-induced enrichment of AGO2, indicating the direct binding of miR-337-3p to *MMP-14* promoter. Knockdown of *AGO2* abolished the miR-337-3p-induced binding of repressive epigenetic markers, accompanying by increased MZF1 enrichment on *MMP-14* promoter. We believe that miR-337-3p/AGO2 complexes may bring in co-repressors such as histone methyltransferases to repress the binding of transcription factor MZF1, which warrants further investigation.

In summary, we have shown that MZF1 is highly expressed and directly binds to the *MMP-14* promoter to facilitate its transcription in gastric cancer. Meanwhile, miR-337-3p is under-expressed in human gastric cancer, and suppresses the transcription of *MMP-14* via epigenetically suppressing the binding of MZF1 to its promoter, resulting in decreased growth, invasion, metastasis, and angiogenesis of gastric cancer cells *in vitro* and *in vivo*. Although MZF1 and miR-337-3p are found to be independent prognostic factors, a larger series of clinical specimens are needed to further explore the cooperative effects of high MZF1 expression and low miR-337-3p levels on the outcome of gastric cancer patients. This study extends our knowledge about the regulation of *MMP-14* at transcriptional level, and suggests that MZF1 and miR-337-3p may be of potential values as novel therapeutic targets for human gastric cancer.

## MATERIALS AND METHODS

### Cell culture

Human gastric cancer cell lines AGS (CRL-1739), SGC-7901, MKN-28 and MKN-45, normal gastric epithelial GES-1 cells, and human endothelial cell line HUVEC (CRL-1730) were obtained from the American Type Culture Collection (Rockville, MD) and Type Culture Collection of Chinese Academy of Sciences (Shanghai, China). Cell lines were authenticated by the provider, used within 6 months after resuscitation of frozen aliquots, and grown in RPMI1640 medium (Life Technologies, Inc., Gaithersburg, MD) supplemented with 10% fetal bovine serum (Life Technologies, Inc.), penicillin (100 U/ml), and streptomycin (100 mg/ml). Cells were maintained at 37°C in a humidified atmosphere of 5% CO_2_ and applied for transfection.

### Gene over-expression and knockdown

Human *MZF1* expression vector was kindly provided by Dr. Jiawei Zhou (Shanghai Institutes for Biological Sciences, Chinese Academy of Sciences, China) [37]. The oligonucleotides encoding shRNAs specific for *MZF1* and *AGO2* (Supplementary Table S5) were subcloned into the *Bam H* I and *Hind* III restrictive sites of GV102 (Genechem Co., Ltd, Shanghai, China). The *MZF1* or *MZF1* shRNA vectors were transfected into cancer cells with Lipofectamine 2000 (Life Technologies, Inc.), and stable cell lines were screened by administration of puromycin (Invitrogen, Carlsbad, CA). The pcDNA3.1 and sh-Scb were applied as controls (Supplementary Table S5).

### Western blot

Cellular protein was extracted with 1× cell lysis buffer (Promega, Madison, WI). Western blot was performed as previously described [15, 20, 38–47], with antibodies specific for MZF1 (Santa Cruz Biotechnology, Santa Cruz, CA), MMP-14 (Abcam Inc, Cambridge, MA), VEGF (Santa Cruz Biotechnology), AGO2 (Cell Signaling Technology, Inc., Danvers, MA), and β-actin (Santa Cruz Biotechnology).

### Real-time quantitative RT-PCR

Total RNA was isolated with RNeasy Mini Kit (Qiagen Inc., Valencia, CA). The reverse transcription reactions were conducted with Transcriptor First Strand cDNA Synthesis Kit (Roche, Indianapolis, IN). Real-time PCR was performed with SYBR Green PCR Master Mix (Applied Biosystems, Foster City, CA) and primers indicated in Supplementary Table S6. The transcript levels were analyzed by 2^−ΔΔCt^ method.

### miRNA prediction and expression detection

miRNA binding sites within the *MMP-14* promoter were analyzed using the algorithm microPIR [48]. The mature miR-337-3p levels were determined using Bulge-Loop^TM^ miRNAs qPCR Primer Set (RiboBio Co. Ltd, Guangzhou, China). After cDNA was synthesized with a miRNA-specific stem-loop primer, the quantitative PCR was performed with the specific primers (Supplementary Table S6). The miRNA levels were normalized as to those of U6 snRNA.

### Over-expression and knockdown of miRNA

According to the pre-miR-337-3p (5′-GAACGGCT TCATACAGGAGTT-3′) sequence documented in the miRNA Registry database [49], oligonucleotides encoding the precursor of miR-337-3p (Supplementary Table S5) were subcloned into pcDNA3.1(−) (Genechem Co., Ltd, Shanghai, China). The plasmids pcDNA3.1 and pcDNA3.1-miR-337-3p were transfected into cancer cells, and stable cell lines were screened by administration of neomycin (Invitrogen, Carlsbad, CA). Anti-miR-337-3p or negative control inhibitors (RiboBio Co. Ltd) were transfected into confluent cells with Lipofectamine 2000 (Life Technologies, Inc.).

### Luciferase reporter assay

Human *MMP-14* promoter luciferase reporter construct was kindly provided by Dr. Jouko Lohi (University of Helsinki, Finland) [11]. Mutation of MZF1 or miR-337-3p binding site was established with GeneTailor^TM^ Site-Directed Mutagenesis System (Invitrogen) and PCR primers (Supplementary Table S5). Dual-luciferase assay was performed as previously described [15, 20, 38–40, 43–47, 50]. For *MMP-14* promoter activity, the luciferase signal was normalized by firefly/*Renilla* ratio.

### Nuclear run-on assay

Nuclear run-on assay was performed based on the incorporation of biotin-16-uridine-5′-triphosphate (biotin-16-UTP) into nascent transcripts as previously described [20, 43, 46, 50]. Briefly, nuclei of 5 × 10^6^ cancer cells were isolated and consequently incubated in reaction buffer containing rNTPs and biotin-16-UTP (Roche, Indianapolis, IN) at 30°C for 45 min. The reaction was stopped by adding RNase-free DNase I, and nuclei were lysed and treated with proteinase K. Total RNA was extracted using Trizol (Invitrogen), and biotinylated nascent RNA was purified using agarose-conjugated streptavidin beads (Invitrogen) for real-time quantitative RT-PCR assay.

### Chromatin immunoprecipitation

ChIP assay was performed according to the instructions of EZ-ChIP kit (Upstate Biotechnology, Temacula, CA) [15, 21, 40, 44, 45, 47, 50], with antibodies for MZF1, AGO2, H3K9me2, H3K27me3, and H3K4me3 (Upstate Biotechnology, Temacula, CA). Lysates were treated with either RNase H (10 U) or RNase A (20 μg) prior to immunoprecipitation. DNA was sonicated into fragments of an average size of 200 bp. Real-time qPCR was performed with SYBR Green PCR Master Mix and primer sets indicated in Supplementary Table S6. The amount of immunoprecipitated DNA was calculated in reference to a standard curve, and the results were normalized to input DNA.

### Cell viability assay

Cancer cells were cultured in 96-well plates at 5×10^3^ cells per well. Cell viability was monitored by the 2-(4,5-dimethyltriazol-2-yl)-2,5-diphenyl tetrazolium bromide (MTT; Sigma, St. Louis, MO) colorimetric assay [50]. All experiments were done with 6–8 wells per experiment and repeated at least three times.

### Soft agar assay

Cancer cells at 5 × 10^3^ per well were mixed with 0.05% Nobel agar (Fisher Scientific, Pittsburgh, PA) in growth medium and plated onto 6-well plates containing a solidified bottom layer (0.1% Noble agar in growth medium). After the incubation of cells for 21 days, the number of cell colonies was counted under the microscope, and the cells were fixed with 100% methanol and stained with 0.5% crystal violet dye [38, 40, 43, 47].

### Cell invasion assay

Matrigel invasion assay was performed using membranes coated with Matrigel matrix (BD Science, Sparks, MD). Homogeneous single cell suspensions (1 × 10^5^ cells/well) were added to the upper chambers and allowed to invade for 24 hrs at 37°C in a CO_2_ incubator. Invaded cells were stained with 0.1% crystal violet for 10 min at room temperature. Quantification of invaded cells was performed according to published criteria [15, 20, 21, 38, 39, 41–47, 51].

### Tube formation assay

Fifty microliters of growth factor-reduced matrigel were polymerized on 96-well plates. HUVECs were serum starved in RPMI1640 medium for 24 hrs, suspended in RPMI1640 medium preconditioned with cancer cells, added to the matrigel-coated wells at the density of 5×10^4^ cells/well, and incubated at 37°C for 18 hrs. Quantification of anti-angiogenic activity was calculated as previously described [15, 20, 38, 40, 41, 43, 46].

### *In vivo* growth and metastasis assay

All animal experiments were approved by the Animal Care Committee of Tongji Medical College (approval number: Y20080290). For the *in vivo* tumor growth studies, 2-month-old male nude mice (n = 5 per group) were injected subcutaneously in the upper back with 1×10^6^ cancer cells stably transfected with empty vector, *MZF1*, or miR-337-3p precursor. One month later, mice were sacrificed and examined for tumor weight and gene expression. The experimental metastasis (0.4×10^6^ tumor cells per mouse, n = 5 per group) studies were performed with 2-month-old male nude mice as previously described [15, 20, 38–40, 43–47].

### Patient tissue samples

Approval to conduct this study was obtained from the Institutional Review Board of Tongji Medical College (approval number: 2011-S085). Fresh specimens of 90 well-established primary gastric cancer cases were obtained from the Department of Surgery, Union Hospital, Tongji Medical College. Their pathological diagnosis was proved by at least two pathologists. The demographic and clinicopathological data of subtotal 50 patients were summarized in Supplementary Table S1. Adjacent gastric mucosa specimens that contained no macroscopic tumor were also obtained, and the non-neoplastic areas were subsequently verified by microscopic histology to be free of tumor infiltration. The fresh tumor and adjacent normal gastric specimens were collected and stored at −80°C until use.

### Immunohistochemistry

Immunohistochemical staining was performed as previously described [15, 20, 38–40, 43–47, 52], with antibodies specific for MZF1 (Santa Cruz Biotechnology; 1:200 dilution), MMP-14 (Abcam Inc; 1:200 dilution), and CD31 (Santa Cruz Biotechnology; 1:200 dilution). The negative controls included parallel sections treated without primary antibody or with rabbit polyclonal IgG (Abcam Inc.). The immunoreactivity in each tissue section was assessed by at least two pathologists. The degree of positivity was determined according to the percentage of positive tumor cells.

### Statistical analysis

Unless otherwise stated, all data were shown as mean ± standard error of the mean (SEM). The χ^2^ analysis and Fisher exact probability analysis were applied to compare the gene expression in cancer tissues. Pearson's coefficient correlation was applied for analyzing the relationship among gene expression. The log-rank test and Cox regression models were used to assess survival difference and hazard ratios. The Bonferroni method was applied to analyze the difference of survival curves. Difference of tumor cells was determined by *t* test or analysis of variance (ANOVA).

## SUPPLEMENTARY MATERIALS FIGURES AND TABLES


