# Supplementary material for: miRNA-337-3p inhibits gastric cancer progression through repressing myeloid zinc finger 1-facilitated expression of matrix metalloproteinase 14
Source: Oncotarget. 2016 May 31;7(26):40314–28. doi: 10.18632/oncotarget.9739 (PMC5130010; doi:10.18632/oncotarget.9739)
Supplement: Supplementary file 1 [file oncotarget-07-40314-s001.pdf]

miRNA-337-3p inhibits gastric cancer progression through repressing myeloid zinc finger 1-facilitated expression of matrix metalloproteinase 14

Supplementary Materials

Supplementary Table S1: MZF1 and MMP-14 expression in human gastric cancer tissues

Clinicopathologic factor	Total	MZF1 expression		MMP-14 expression	
	n **(%)**	***n*** **(%)**	***P*-value**	***n*** **(%)**	***P*-value**
**Age (years)**					
≤ 60	26 (52.0)	16 (61.5)	0.817	18 (69.2)	0.902
> 60	24 (48.0)	14 (58.3)		17 (70.8)	
**Sex**					
Male	35 (70.0)	21 (60.0)	1.000	24 (68.6)	0.736
Female	15 (30.0)	9 (60.0)		11 (73.3)	
**Size (diameter)**					
≤ 6 cm	33 (66.0)	20 (60.1)	1.000	23 (69.7)	0.948
> 6 cm	17 (34.0)	10 (58.8)		12 (70.6)	
**Laurén classification**					
Intestinal type	29 (58.0)	17 (58.6)	0.418	20 (68.9)	0.851
Diffuse type	21 (42.0)	13 (61.9)		15 (71.4)	
**Gastric wall invasion**					
T1/T2	18 (36.0)	2 (11.1)	< 0.001	6 (33.3)	< 0.001
T3/T4	32 (64.0)	28 (87.5)		29 (90.6)	
**Lymph node metastasis**					
Negative	18 (36.0)	4 (22.2)	< 0.001	5 (27.8)	< 0.001
Positive	32 (64.0)	26 (81.3)		30 (93.8)	
**Distant metastasis**					
Negative	38 (76.0)	19 (50.0)	0.029	23 (60.5)	0.009
Positive	12 (24.0)	11 (91.7)		12 (100.0)	
**TNM stage**					
I/II	17 (34.0)	3 (17.6)	< 0.001	5 (29.4)	< 0.001
III/IV	33 (66.0)	27 (81.8)		30 (90.9)	

MZF1, myeloid zinc finger 1; MMP-14, matrix metalloproteinases 14; TNM, tumor-node-metastasis.

Supplementary Table S2: Correlation between the expression of MZF1 and MMP-14

	MZF1 expression			
	Low	High	*R*-value	*P*-value
**MMP-14 expression**				
Low	18	2	0.500	< 0.001
High	12	18		

MZF1, myeloid zinc finger 1; MMP-14, matrix metalloproteinases 14; Pearson's correlation coefficient was applied to determine the expression correlation.

Supplementary Table S3: Comparison of survival curves of gastric cancer patients

Comparison	Chi-square	Bonferroni *P*-value
MZF1-low v.s. MZF1-high	56.33	$< 1.0 \times 10^{-10}$
MZF1-low v.s. MMP-14-low	1.49	1
MZF1-low v.s. MMP-14-high	30.61	1.6×10^{-7}
MZF1-low v.s. miR-337-3p-low	46.26	$< 1.0 \times 10^{-10}$
MZF1-low v.s. miR-337-3p-high	0.02	1
MZF1-high v.s. MMP-14-low	44.17	$< 1.0 \times 10^{-10}$
MZF1-high v.s. MMP-14-high	5.36	0.1029
MZF1-high v.s. miR-337-3p-low	0.38	1
MZF1-high v.s. miR-337-3p-high	55.72	$< 1.0 \times 10^{-10}$
MMP-14-low v.s. MMP-14-high	30.41	1.8×10^{-7}
MMP-14-low v.s. miR-337-3p-low	37.09	$< 1.0 \times 10^{-10}$
MMP-14-low v.s. miR-337-3p-high	1.27	1
MMP-14-high v.s. miR-337-3p-low	2.71	0.4992
MMP-14-high v.s. miR-337-3p-high	31.24	1.1×10^{-7}
miR-337-3p-low v.s. miR-337-3p-high	46.35	$< 1.0 \times 10^{-10}$

MZF1, myeloid zinc finger 1; MMP-14, matrix metalloproteinases 14; Bonferroni method was applied for the comparison of survival curves.

Supplementary Table S4: Univariate and multivariate analysis of prognostic factors in gastric cancer patients

Variables	*n*	Univariate analysis		Multivariate analysis		
		Mean ± SEM (months)	*P*-value	Hazard ratio	95% CI	*P*-value
**Age (years)**						
≤ 60	26	35.4 ± 4.9	0.902	1.017	0.482–3.143	0.765
> 60	24	36.7 ± 5.1				
**Sex**						
Male	35	37.0 ± 4.2	0.872	1.397	0.427–3.465	0.675
Female	15	33.3 ± 6.6				
**Size (diameter)**						
≤ 6 cm	33	37.2 ± 4.2	0.896	1.807	1.311–5.162	0.467
> 6 cm	17	33.9 ± 6.4				
**Laurén classification**						
Intestinal type	29	41.2 ± 4.3	0.146	1.596	0.352–4.233	0.545
Diffuse type	21	28.9 ± 5.7				
**Gastric wall invasion**						
T1/T2	18	64.7 ± 0.2	< 0.001	2.218	1.682–6.924	0.332
T3/T4	32	18.9 ± 2.5				
**Lymph node metastasis**						
Negative	18	61.5 ± 0.3	< 0.001	2.018	1.339–5.756	0.431
Positive	32	20.7 ± 2.5				
**Distant metastasis**						
Negative	38	44.8 ± 3.6	< 0.001	2.052	1.976–5.513	0.002
Positive	12	8.1 ± 0.5				
**TNM stage**						
I/II	17	64.7 ± 0.2	< 0.001	2.722	1.976–6.713	0.005
III/IV	33	20.5 ± 2.8				
**MZF1 expression**						
Low	30	52.3 ± 3.4	< 0.001	3.516	1.537–8.942	0.018
High	20	11.6 ± 1.3				
miR-337-3p expression						
Low	22	59.3 ± 2.8	< 0.001	0.393	0.128–0.705	0.015
High	28	20.0 ± 3.1				
**MMP-14 expression**						
Low	20	13.6 ± 2.4	< 0.001	2.319	1.275–6.334	0.028
High	30	53.5 ± 3.3				

MZF1, myeloid zinc finger 1; MMP-14, matrix metalloproteinases 14; TNM, tumor-node-metastasis; Log-rank test and Cox regression model were applied for univariate and multivariate analysis. *n*, number of patients; SEM, standard error of the mean; 95% CI, 95% confidence interval.

Supplementary Table S5: Oligonucleotide sets used for constructs, inhibitors and short hairpin RNAs

Oligo Set	Sequences
Pre-miR-337-3p	5′-TGCTGCTCCTATATGATGCCTTTCTTCGTTTTGGCCACTGACTGACGAAGAAAGGCATC ATATAGGAG-3′(sense);
	5′-CCTGCTCCTATATGATGCCTTTCTTCGTCAGTCAGTGGCCAAAACGAAGAAAGGCATCA TATAGGAGC-3′(antisense)
Pre-miR-NC	5′-TGCTGAAATGTACTGCGCGTGGAGACGTTTTGGCCACTGACTGACGTCTCCACGCAG TACATTT-3′(sense);
	5′-CCTGAAATGTACTGCGTGGAGACGTCAGTCAGTGGCCAAAACGTCTCCACGCGCAGT ACATTTC-3′(antisense)
pGL3-MMP14 mut (ΔMZF1)	5′-CTGGGGCTTTCACGGAGGAGAGGCTGTGGGAGAAGG-3′(sense);
	5′-CTCCTCCGTGAAAGCCCCAGTGCCCTCCTTTCCTGGT-3′(antisense)
pGL3-MMP14 mut (ΔmiR-337-3p)	5′-CTGGGGCGGGGACGGAGGAGCAATTGTGTTCAAAGGGAGGGA-3′(sense);
	5′-CTCCTCCGTCCCCGCCCCAGTGCCCTCCTT-3′(antisense)
Anti-NC	RiboBio
Anti-miR-337-3p	RiboBio
sh-Scb	5′-CCGGGCGAACGATCGAGTAAACGGACTCGAGTCCGTTTACTCGATCGTTCGCTTTTT-3′ (sense);
	5′-AATTCAAAAAGCGAACGATCGAGTAAACGGACTCGAGTCCGTTTACTCGATCGTTCGC-3′ (antisense)
sh-MZF1-1	5′-CCGGCCACCAGAGCACCAAGCTCATCTCGAGATGAGCTTGGTGCTCTGGTGGTTTTTG-3′ (sense);
	5′-AATTCAAAAACCACCAGAGCACCAAGCTCATCTCGAGATGAGCTTGGTGCTCTGGTGG-3′ (antisense)
sh-MZF1-2	5′-CCGGCCGTTGCGATGTATGTGGCAACTCGAGTTGCCACATACATCGCAACGGTTTTTG-3′ (sense);
	5′-AATTCAAAAACCGTTGCGATGTATGTGGCAACTCGAGTTGCCACATACATCGCAACGG-3′ (antisense)
sh-AGO2-1	5′-CCGGGCACAGCCAGTAATCGAGTTTCTCGAGAAACTCGATTACTGGCTGTGCTTTTTG-3′ (sense);
	5′-AATTCAAAAAGCACAGCCAGTAATCGAGTTTCTCGAGAAACTCGATTACTGGCTGTGC-3′ (antisense)
sh-AGO2-2	5′-CCGGCGTCCGTGAATTTGGAATCATCTCGAGATGATTCCAAATTCACGGACGTTTTTG-3′ (sense);
	5′-AATTCAAAAACGTCCGTGAATTTGGAATCATCTCGAGATGATTCCAAATTCACGGACG-3′ (antisense)

Pre-miR-NC, negative control pre-miRNA; MMP-14, matrix metalloproteinases 14; MZF1, myeloid zinc finger 1; Anti-NC, negative control inhibitor; sh-Scb, scramble short hairpin RNA; AGO2, argonaute 2.

Supplementary Table S6: Primer sets used for qRT-PCR, nuclear run-on, and ChIP

Primer set	Primers	Sequence	Product size (bp)	Application
MMP-14	Forward	5′-GCCTTCTGTTCCTGATAA-3′	225	qRT-PCR
	Reverse	5′-CCATCCTTCCTCTCGTAG-3‘		nuclear run-on
VEGF	Forward	5′-ATGACGAGGGCCTGGAGTGT-3′	226	qRT-PCR
	Reverse	5′-CATTTACACGTCTGCGGATCT-3′		
MZF1	Forward	5′-CTGAAACTGAGCCTCCAACTCC-3′	232	qRT-PCR
	Reverse	5′-CCAGTCTTGCTGTGGGGAAA-3′		
β-actin	Forward	5′-ATCTACGAGGGGTATGCC-3′	227	qRT-PCR
	Reverse	5′-TAGCTCTTCTCCAGGGAG-3′		
miR-337-3p	Forward	RiboBio		qRT-PCR
	Reverse	RiboBio		
U6	Forward	RiboBio		qRT-PCR
	Reverse	RiboBio		
MMP-14 set 1	Forward	5′-TCAAGCCACTCAGAATATGC-3′	170	ChIP
(–326/–157)	Reverse	5′-ACCAAGAACTGGAAGGAAAA-3′		
MMP-14 set 2	Forward	5′-CAACCAGGAAAGGAGGGC-3′	191	ChIP
(–122/+69)	Reverse	5′-TCGGCTTGGAGTTAAAGG-3′		

MMP-14, matrix metalloproteinase 14; VEGF, vascular endothelial growth factor; MZF1, myeloid zinc finger 1.

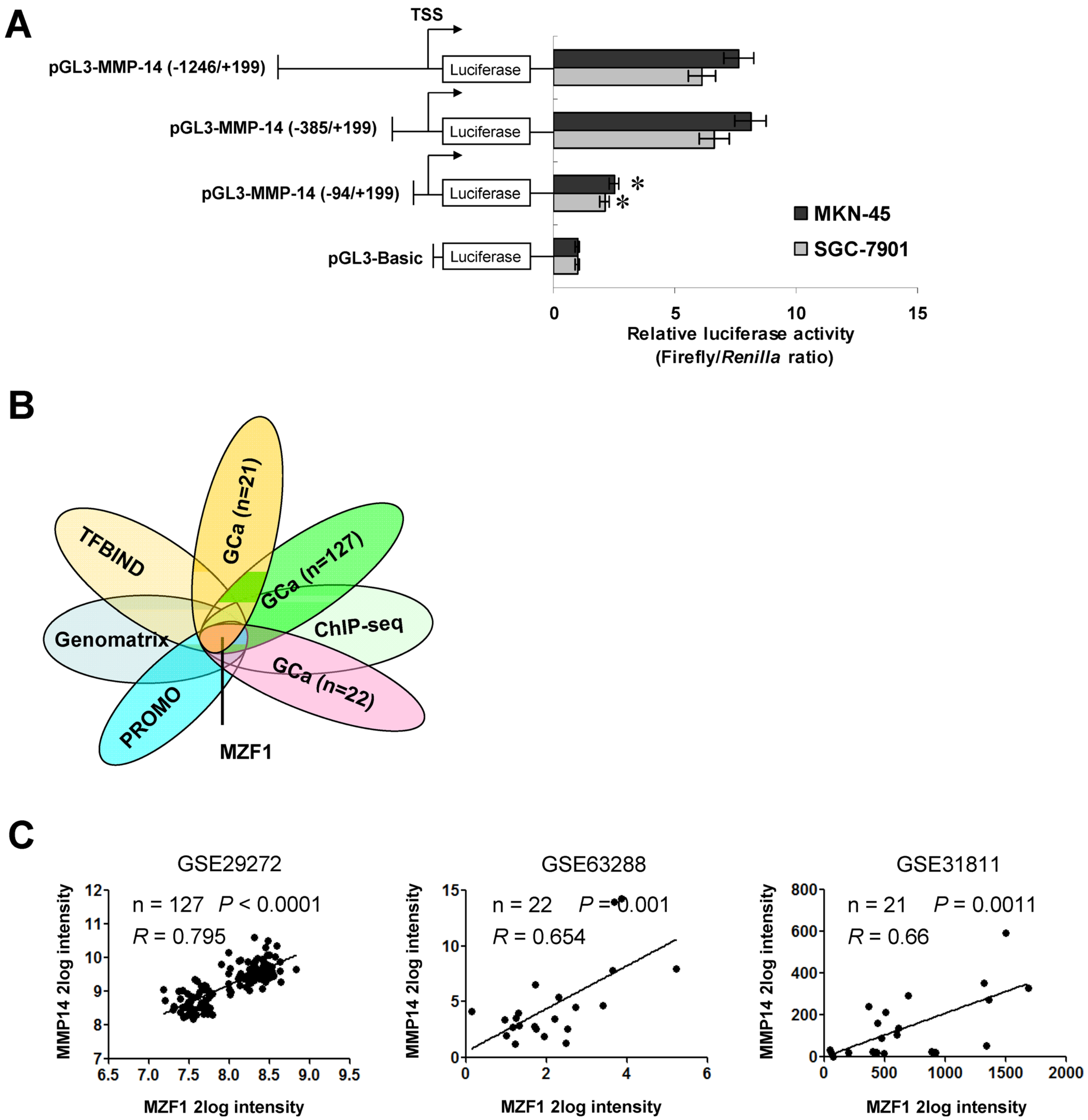

Supplementary Figure S1: Determining the transcription factor crucial for MMP-14 expression. (**A**), dual-luciferase assay showing the activity of different *MMP-14* promoter fragments in gastric cancer cells. (**B**), over-lapping analysis revealing MZF1 as a crucial transcription factor binding to the *MMP-14* promoter region (chr14:23305444-23305733). (**C**), the correlation between MZF1 and MMP-14 levels in gastric cancer cohorts derived from Gene Expression Omnibus (GEO) datasets. **P* < 0.01 vs. pGL3-MMP-14 (–1246/+199).

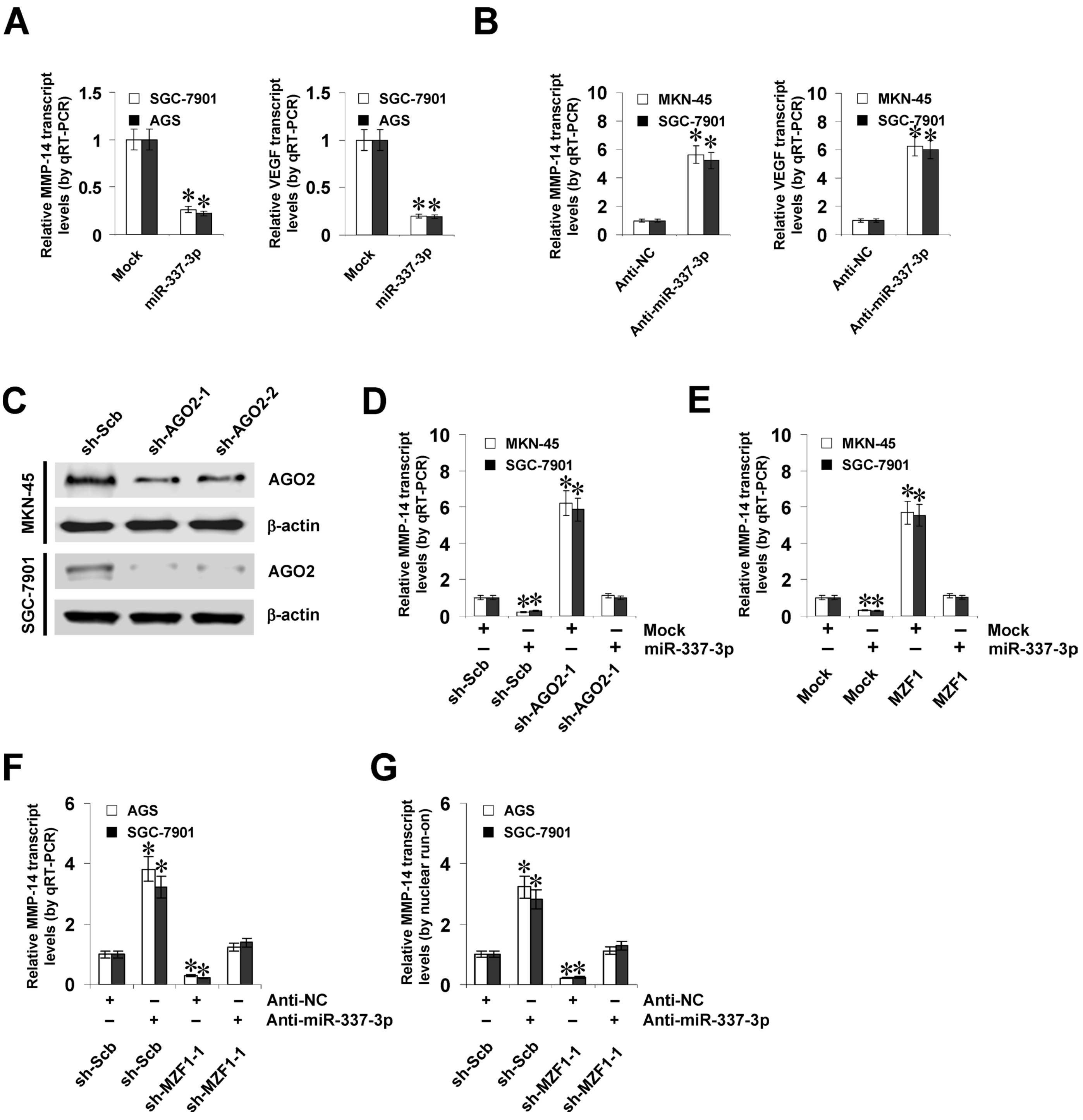

Supplementary Figure S2: Expression of MMP-14 and VEGF in gastric cancer cells. (**A** and **B**), real-time quantitative RT-PCR showing the transcript levels of *MMP-14* and *VEGF* in gastric cancer cells transfected with empty vector (mock), miR-337-3p precursor, anti-NC (100 nmol/L), or anti-miR-337-3p inhibitor (100 nmol/L). (**C**), western blot indicating the AGO2 expression in gastric cancer cells transfected with scramble shRNA (sh-Scb) or AGO2 shRNA (sh-AGO2). (**D** and **E**), real-time quantitative RT-PCR showing the transcript levels of *MMP-14* in gastric cancer cells stably transfected with mock or miR-337-3p precursor, and those co-transfected with sh-Scb, sh-AGO2, mock, or MZF1. (**F** and **G**), real-time quantitative RT-PCR and nuclear run-on assays indicating the transcript levels and nascent transcription of *MMP-14* in gastric cancer cells transfected with anti-NC (100 nmol/L) or anti-miR-337-3p inhibitor (100 nmol/L), and co-transfected with sh-Scb or sh-MZF1. *$P < 0.01$ vs. mock, anti-NC, mock+sh-Scb, or anti-NC+sh-Scb.

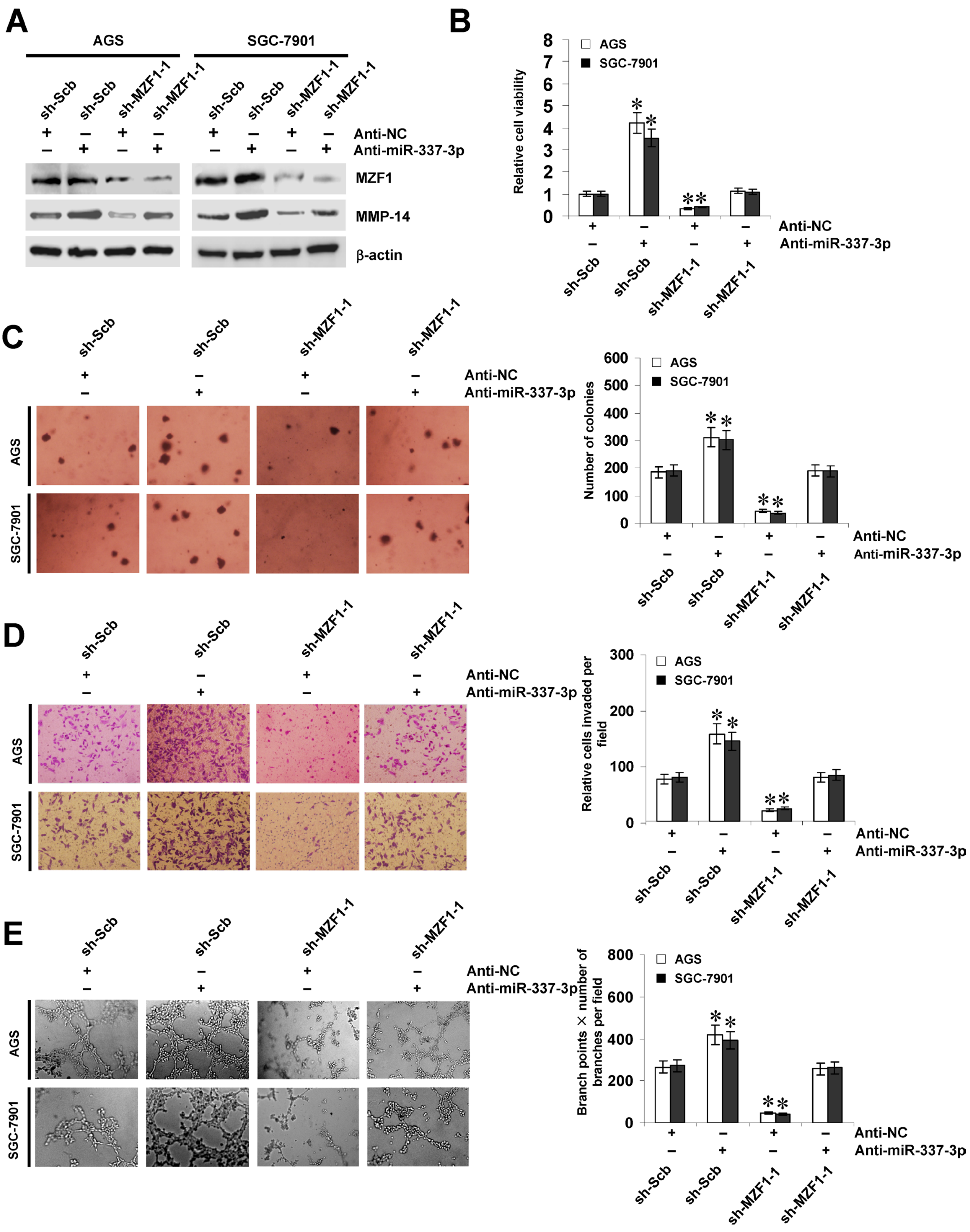

Supplementary Figure S3: Effects of miR-337-3p and MZF1 on the growth, invasion, and angiogenesis of gastric cancer cells. Gastric cancer cells were transfected with anti-NC (100 nmol/L) or anti-miR-337-3p inhibitor (100 nmol/L), and co-transfected with sh-Scb or sh-MZF1. Western blot assay (**A**) showing the expression of MZF1 and MMP-14. MTT colorimetric (**B**), soft agar (**C**), matrigel invasion (**D**), tube formation (**E**) assays indicating the cellular viability, growth, invasion, angiogenesis capability, respectively. **P* < 0.01 vs. anti-NC+sh-Scb.

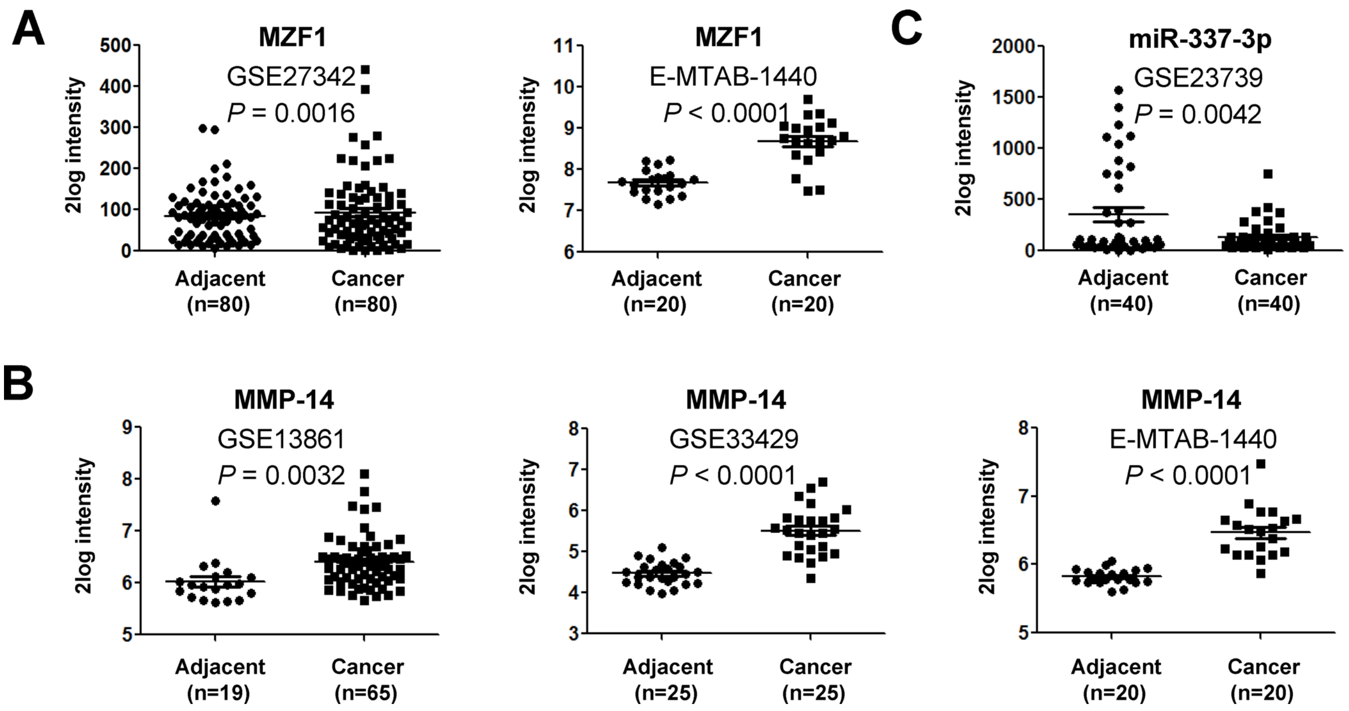

Supplementary Figure S4: Expression of MZF1, MMP-14, and miR-337-3p in public databases. (**A**, **B**, and **C**), the MZF1, MMP-14, and miR-337-3p expression in gastric cancer and adjacent normal tissues derived from Gene Expression Omnibus (GEO) and ArrayExpress (http://www.ebi.ac.uk/arrayexpress) datasets.
